# A Historical Review of Diachrony and Semantic Dimensions of Trace in Neurosciences and Lacanian Psychoanalysis

**DOI:** 10.3389/fpsyg.2017.00734

**Published:** 2017-06-23

**Authors:** Carolina Escobar, François Ansermet, Pierre J. Magistretti

**Affiliations:** ^1^Agalma FoundationGeneva, Switzerland; ^2^Centre for Psychiatric Neurosciences, Lausanne University HospitalLausanne, Switzerland; ^3^Department of Psychiatry, Faculty of Medicine, University of GenevaGeneva, Switzerland; ^4^Brain Mind Institute, Swiss Federal Institute of Technology in LausanneLausanne, Switzerland; ^5^Division of Biological and Environmental Sciences and Engineering, King Abdullah University of Science and TechnologyThuwal, Saudi Arabia

**Keywords:** trace, diachrony, semantics, reconsolidation, syntax, reassociation, *Nachträglichkeit* (deferred action), engram

## Abstract

Experience leaves a trace in the nervous system through plasticity. However, the exact meaning of the *mnesic trace* is poorly defined in current literature. This article provides a historical review of the term trace in neuroscience and psychoanalysis literature, to highlight two relevant aspects: the diachronic and the semantic dimensions. There has been a general interest in diachrony, or a form of evolution of the trace, but its indissociable semantic dimension remains partially disregarded. Although frequently implied, the diachronic and semantic dimensions of the trace are rarely clearly articulated. We situate this discussion into the classical opposition of syntax, or rules of inscription of the trace in the nervous system, and semantics, or the content of the trace, which takes into consideration the attempt of the human being to build coherence. A general observation is that the study of the term trace follows trends of the thought of the given epoch. This historical analysis also reveals the decay of the idea that the trace is reliable to the experience. From the articulation between neurosciences and psychoanalysis in a historical perspective, this review shows that the trend is to consider trace as a production of the subject, resulting in a permanent rewriting in an attempt to give meaning to the experience. This trend is becoming increasingly evident in light of recent research in neurosciences and psychoanalysis.

## Introduction

From a psychoanalytic clinical perspective, the inscription of *enjoyment* (*jouissance* in French), or a transgressive quantity of psychic energy beyond pleasure, is much discussed: “for the being who speaks and demands, the body fails the *enjoyment* inscription” (Laurent, 2016, p. 15). From a neuroscientific or a materialistic perspective, this statement is contradicted by experimental work carried out in the 100 years. Indeed, between neuroscience and psychoanalysis, there is an epistemological gap, which this article aims to explore. Herewith, we focus on the definition of the inscription of the mnesic trace from a historical review of neuroscience and psychoanalysis literature, and from current research in each field.

A first general observation is that the different notions of “trace,” on both neurosciences and psychoanalysis, follow the trends of thinking of the given epoch and this is the way in which literature on “trace” has been organized. The first section between empiricism and humanism, the second on cybernetics, the third on neurosciences (that extends into today), and the last section on phenomenology. It is not, however, a history of the philosophy of the notion of “trace”: neither psychoanalysis, nor cybernetics nor neurosciences are here considered as philosophical theories. Moreover, the opposition between syntax and semantics provides in turn a framework to group the different trends of thought: empiricism, cybernetics provides a syntactic grasp of the trace, given that it aims to describe the rules of inscription. Conversely, humanism and phenomenology provide a definition of trace with a semantic consideration.

The term “trace” is referred to by Plotinus who described it as a passive condition produced by events. Later on, Pierre Nicole, an empiricist philosopher close to Locke (1632–1704), used the term “trace” to propose a theological idea of the tabula rasa: sensory experiences leave a trace in the mind, and this is the primary way of knowing oneself. Given that it is related to the object and not to the perceiving subject, perception “hits” our senses.^1^ The trace is an impression remaining in the mind; according to empiricists, it is related to *primary qualities*, or a property of the object independent of the observer. In a subsequent second stage, concepts produced by sensations, or *secondary qualities*, are built on the basis of traces resulting from experience, but they are not reliable knowledge, as they are less in correspondence with the world than *primary qualities*.

At that time, trace was considered as passively accepted by the subject. The construction of meaning was not involved, and it was only considered a “pure” trace in its first inscription. As developed below, this definition had a major influence in the later theoretical developments. However, these defined features of trace have been questioned since the late XIXth century; other important considerations have contributed to understanding memory mechanisms, which have in turn raised unresolved issues. Indeed, two axes are recurrent in the literature: the evolution through lifespan of the trace, or diachrony, and the role of the semantic dimension, or the construction of meaning by the subject. Although the presence of the latter is not always explicit in neuroscientific work, it appears frequently whether studies refer to human memory.

Unlike the definition proposed by empiricists (*primary* and *secondary qualities*), current research on reconstruction of trace reveals an innumerable quantity of possible stages of trace, given its endless possible reassociations (Sara, 2000; Dudai, 2012). Indeed, since 1900 with work by Müller and Pilzecker, researchers have been interested in the interference phenomenon, particularly retroactive interference: experiences occurring immediately after the event may interfere in the inscription of the experience in the nervous system. The continuation of Müller and Pilzecker’s work gave rise to the *consolidation model*, a process of stabilization of memory beginning immediately after its acquisition. Moreover, in the last two decades, research has focused on another mode of retroactive interference, whereby retrieved traces are susceptible to a transient phase allowing amnesic agents or new traces to update the behavioral expression of the memory (following animal and human studies). This has been called the *reconsolidation model*, and it is implicated in what is known as the “diachronic” dimension of trace (Ansermet and Magistretti, 2010), on the basis of the Saussurian distinction between synchrony and diachrony of languages.^2^

Inspired by psychoanalytical praxis, the diachrony involved in memory reconsolidation has been related to the discontinuity between experience and its inscription through the production of new traces resulting from trace reassociation (Alberini et al., 2013). The trace arising from this process is then not over-determined by the first inscription. Rather, it would be unpredictable and favored by the openness of the plastic features of the brain. This discontinuity is related to a form of autopoiesis of the subject, via the creation of new traces from inaugural traces (Ansermet et al., 2014).

Nevertheless, the diachronic dimension of the trace does not map the reconsolidation model insofar as it is delimited by the computational theory of mind, as defined by Pinker (1997, p. 25): “The computational theory of mind says that beliefs and desires are *information* incarnated as configurations of symbols”. Diachrony is understood rather, in articulation with the semantic dimension, as an overhaul of the trace given the subject’s impossibility of processing the meaning of the trace, when there are not symbols. Therefore, diachrony does not concern the treatment of the information, but the process occurring when there is not enough information; it is related to a construction following the inaccessibility of an absolute meaning of the trace.

This semantic dimension of the trace is understood in this paper as the imaginary transcendence of the relation between the sign and its signification; the meaning gives the impression that a sign can occupy an assigned place, as if the signifier and the signified were linked. However, the relation of meaning is arbitrary, and the sound image (the signifier) seems to make an unambiguous reference to the object (the signified). On the one hand, the semantic dimension of the trace implies an integration of the experience, and on the other hand, of the social convention. In this case, the meaning is an illusion, because the full information of both aspects is inaccessible for the subject. Semantics is understood here as a construction: the subject cannot process all the environmental information (Raichle, 2010) as well as the social network evoke other significations, that the subject knows unknown for him. Already in 1953–1954, Jacques Lacan underlined this polyvalence of semantics: “every semanteme refers to the whole of the semantic system, to the polyvalence of its usages. Moreover, for everything which properly speaking pertains to language, in as much as it is human, that is to say utilizable in speech, the symbol is never univocal. Every semanteme always has several meanings” (Lacan, 1991 [1953–1954], p. 248). According to Lacan, the meaning is neither understood as intrinsic to the surrounding world, nor intrinsic to the trace itself. It has to be created by the subject within his social network (we restricted our research to semantics and diachrony, although intersubjectivity deserves more attention regarding creation of the meaning and the construction of the trace). The meaning is neither defined as the content or proposition, nor near to the intentionality issue, as it is not a feature of the consciousness; on the contrary, it is a product of the unknown. It may seem paradoxical, the meaning is built on no enough information, but precisely, the meaning does not have an information status.

The two dimensions of diachrony and semantics may be related to two approaches highlighted by Goldstein (2011): the *physiological approach* that determines the mechanism by which the stimulus is represented by neurons arousal, which studies diachrony through the reconsolidation model, and the *mental approach*, which encompasses semantics through studying how a stimulus or an experience is represented in the mind. In the same direction, Baddeley et al. (2002) distinguishes between two paradigms in the investigation of the human memory, the one inspired since 1885 by Ebbinghaus’ well controlled experiments, and the other inspired since 1883 by Galton and then by Bartlett around 1932, who explored memory with rich and meaningful daily tasks.

In this way, maybe due to the decline of dualistic philosophical proposals and the general favoring of strict materialistic approaches, which goes hand in hand with the syntactic approaches of the brain, semantic terminology has become almost obsolete. Nevertheless, this dimension is frequently implicit in experimental literature. This is why in a historical perspective we address the history of the term “trace” and focus on these two ontological aspects of diachrony and semantics, and on their relationship without losing sight the trends of thought framework.

## Empiricism and Humanism: Trace, Mneme, Engram, Mark Left by Perception, Neuronal Assemblies and Deferred Action (Nachträglichkeit)

In recent neuroscientific literature, the notions of “trace” and “engram” are often interchangeable (Ryan et al., 2015), namely, “the engram is approximately equivalent to the mnesic trace” (Tonegawa et al., 2015, p. 918). However, these two terms have a distinct history. As mentioned above, the term “trace” was referred to by Plotinus as a passive result of the event or experience: “the trace [the perception] keeps of the event is not a memory; it is a condition, something passively accepted” (Plotinus, 2013 [263–268 AD], p. 284). Surprisingly, trace and memory were already distinguished at the time.

Along the same lines, the empiricist Pierre Nicole used the term “trace” to support the tabula rasa thesis: the primary way to access knowledge is the sensory experience, which leaves a trace in the mind. The trace is related to the object and not to the perceiving subject, which is why he states that perception “hits” our senses.^3^ It is an impression remaining in the mind, related to *primary qualities*, or a property of the object independent from the observer. The second stage of the trace is called *secondary quality*, in that concepts are produced by sensations. They are built on the basis of traces resulting from experience, which are not reliable knowledge given that they are not a result from the direct contact with the world.

Conversely, the term “engram” coined by Richard Semon in 1904 was derived from his “engraphic effect” that references its Greek etymology γράμμα, or something in its written form. Yet, Semon was more specific. To name a “nervous irritable tissue,” he used the term “mneme,” instead of “trace” or “engram.” The mneme is supposed to directly respond to perception (Semon, 1921, p. 12). Thus, on the one hand, “mneme” is the first indication of a latent and sustainable modification of the nerve tissue produced by a stimulus, and on the other hand “engram” is relatively similar to the current usage in neurosciences: both terms, “engram” and “trace,” are currently related to cerebral plasticity and memory consolidation (Dudai et al., 2015).

Besides the “engraphy law,” in 1921 Semon described an “ecphory law,” meaning the awakening of the engram from its latent state (Dudai, 2002). The idea is close to what is nowadays called memory recovery. Indeed, other theories have recently evoked the importance of considering not only the “writing,” but also, the “reading” of the trace, respectively, the Multiple Trace Model and the Reconsolidation Model. These models highlight this inextricable feature in trace theory (see below), which may be related to diachrony in that of it hints the role of trace reactivation in other forms taken by the trace through its expression.

Parallel to empiricism, associationism^4^ began with Locke, around 1690. In this same direction, James (1910 [1890]) proposed a distinction between “primary memory” and “secondary memory,” the latter being a duplicate of the first. This thesis recalls the empiricists’ *primary* and *secondary qualities*, with the difference that for this author secondary memory is related to the fact that “the phenomena of memory are among the simplest and most immediate consequences of the fact that our mind is essentially an associating machine” (James, 1983 [1896], p. 123).

Associationism also influenced Sigmund Freud. Since his first writings, he was inspired by empiricism and associationism, yet with clinical and humanistic^5^ inspirations (Tauber, 2009): “[A memory] even if it has not been abreacted, enters the great complex of associations, it comes alongside other experiences, which may contradict it, and is subjected to rectification by other ideas” (Freud and Breuer, 1981 [1893], p. 9). In the same period, in the “Project for a Scientific Psychology” (1895), he attempted to reproduce the naturalistic point of view of his time: in a psychophysiological perspective in this text, Freud defined the “trace” as the result of facilitation (*bahnung* in German) in the neuron contact barriers in direct quantitative response with the quantitative energy of the stimulus. This description is in line with current notions: in 1897, Charles Sherrington first used the term “synapse.”

Once again likely inspired from the *primary* and *secondary qualities*, in a famous letter sent to his friend Fliess in 1986 (Freud, 1998 [1887–1904]), Freud used the term *Wahrnehmungszeichen*, sign (*Zeichen*) of perception (*Wahrnehmungen*), to specify the first stage of the trace, which was a perception mark always unconscious and linked to other traces by association through the simultaneity of presentation^6^.

Although Freud’s theory was close to associationism, it had his own variations. According to Freud, memory remains mostly unconscious, so human beings are only conscious of a small portion of their memories, which are made of traces. In “Project for a Scientific Psychology” (Freud, 1953 [1895]), the concept of “trace” becomes more ontological than epistemological. In order to become conscious, the trace has to be “tamed,” says Freud, meaning that the trace has to follow retranscriptions and rearrangements in relation to novel circumstances. Despite Freud’s commitments to positivism, major differences between Freud and the empiricist tradition are drawn: if the traces are *correctly* rewritten, the best way to know one’s environment and oneself is through thinking. As such, Freud’s definition of retranscriptions hints at the current understanding of reassociation, an evolution of the trace associations (to other traces) through time (Ansermet and Magistretti, 2010), but also takes into consideration the content of the trace.

Within the same text, Freud deepens the definition of the trace, considering the relationship between trace and satisfaction. The subject, submitted to the pleasure principle^7^, attempts to keep a minimum level of excitation. Thereafter, the mark left by perception, or *Wahrnehmungszeichen* in the Freudian terminology, becomes the juncture between language and neuronal excitation. It aims to reach a homeostatic state, which mitigates the arousal (for more details see Ansermet and Magistretti, 2004).

Also in accordance to the pleasure principle, Freud defends that consciousness may be influenced from facilitated neural pathways related to the subject’s satisfactory past experiences in order to rapidly decrease the excitation^8^. It is a general trend toward an activation of the pathways related to past experiences having diminished the displeasure. As such, these facilitations maintain an important *hallucinatory*^9^ component in consciousness, related to trace reactivation that competes with reality, in accordance with the pleasure principle.

The psychoanalyst Jacques Lacan supports this position. In his terms, Freud is essentially saying that the subject is “suturing a hole” when he states that the subject builds his reality according to the pleasure principle. That is, the subject is facing a no inscription of the trace, namely in Freudian terms, it is a *wishful activation*. This hole represents a lack of satisfaction, given that the attempts to reach it are always compared to the inaugural moments of satisfaction, which are related to *enjoyment*. For Lacan, the secondary traces built on the basis of the inaugural trace are indicators, or masks of the loss of satisfaction, related to the first traces (Lacan, 2012 [1967–1968]). It is how we understand Laurent’s statement: “for the being who speaks the body fails the *enjoyment* inscription” (Laurent, 2016). In a Lacanian psychoanalysis, one may say it is why the subject continues to try to inscribe the trace.

The most heuristic Freud’s statements had to wait until Lacan’s insights. But despite the delay in understanding Freud’s proposal, it is a turning point in the evolution of the concept of trace. Not only is the subject unable to properly represent his world, on the basis of successive efforts to find the original trace of satisfaction, he also participates in the construction of the trace since the loss of the original trace. As such, the inclusion of pleasure introduces the major role of trace content in the creation of new traces during the subject’s lifetime, as well as the concept of *retranscriptions* introduces diachrony.

Later, Freud’s writings become more psychological than physiological. Through trace construction, the subject builds a partially fictional history from which the subject may find a relative freedom from original traces (Freud, 1964 [1938]). It becomes particularly relevant in case of traumatism. This idea highlights the efforts of the human mind in building a coherent and meaningful representation of the world. However, it also highlights a paradox of the trace (Ansermet et al., 2014), between experience and construction, as a result of the trace construction in the attempt to invest the meaning. As such, any concern about the reliability of the perception is left behind, the mark left by the experience is not exempt from subject perceiving.

Freud used another term related to trace. It is “deferred action” (*Nachträglichkeit* in Freud’s German), which is associated this time to the overhaul of the traumatism. According to Freud, the traumatism has two phases, one of no inscription and another of retroactive traumatism reconstruction or “deferred action.” In the deferred action theory, a second scene retroactively gives a signification of trauma to a previous scene, which could not be associated before to other traces. Thus, deferred action is a specific form of trace reassociation with retroactive effect over the signifiers related to a prior traumatism. This conception is in continuity with the idea of *Wahrnehmungszeichen*; the experience leaves a mark that is not a trace because it is not linked with other traces. The second scene is generally harmless, but acts as a cued recall, by associative features that rekindle the first scene giving it a traumatic signification.

In summary, the associationism contributed to a syntactic description of the trace in that it provides an explanation of the rules of trace inscription, and it continues to inspire neuroscientific experimentation. Indeed, Hebb (1949) consolidated the idea that simultaneous activation of cell groups makes the group a “neuronal assembly”: persistent activity in one cell promotes activation of another cell in the same group^10^. More specifically, the trace is described as an increase of connective strength among populations of interconnected neurons occurring by repeated cell co-activation. Hence, “neuronal assemblies” is a nowadays widely accepted term suggesting a mechanism, or rules of inscription of the “trace” and “engram.” Thus, this is a definition at a syntactic level in an empiricist and associationist line.

## The Cybernetic Theory of the Trace with the Modal Model: Consolidation, Learning and Long Term Memory

Under the influence of prevailing positivism in the late XIXth century and until nowadays, the syntactic and physiological trace description continued a research line leading to major neuroscientific experimental advances. Thus, in 1900 Müller and Pilzecker studied the role of temporal conditions in learning, the process is termed “consolidation.” In their experiment, participants were divided into “immediate” and “delay” groups, and asked to learn two lists of nonsense syllables. The “delay group” had a 6 min’ interval between memorizing the two lists. These subjects remembered 48% of the syllables, compared with 28% in the “immediate group.” The authors concluded that a phenomenon in which a perseverating neural process was maintained until “a permanent memory structure was formed” (Lewis, 1979). Their work has been inspirational and has had a major influence on the study of memory, by strengthening the position that memory is static once fixed.

In the same positivist direction, in the 1940s there was a growing enthusiasm for cybernetics, the study of transmission and processing of information (in humans, other animals or machines, in equal measure). Thus, new terminology such as “learning” began to appear in scientific literature as an equivalent of “memory.” Based on Atkinson and Shiffrin’s “Modal Model” (1968), sensory memory (SM), short-term memory (STM), and long-term memory (LTM) relayed the terms “trace” and “engram” to help exclude philosophical allusions in positivist science. According to computational models, the nervous system receives an input that is transformed, at first into SM, later becoming STM, and then finally, through consolidation, LTM.

This theory is based on experiments showing the fragility and instability of recently acquired learning in the same vein as Muller and Pilzecker’s experiment: learning strengthens over time and becomes more permanent. In this manner, this theory explains why after electroconvulsive therapy or cerebral concussion the patient may present a retrograde amnesia, and forgets recent experiences for a few seconds or minutes preceding the shock, without forgetting what one experienced several months or years before the shock (Squire, 1986). Therefore, consolidation theory underlines the neuronal and cognitive mechanisms that fix the mnesic trace according to a temporal window, or when transferred from STM to LTM (Atkinson and Shiffrin, 1968).

The term LTM is still widely used in memory theorization, while the term STM has been replaced by *working memory.* This term, credited by Baddeley and Hitch (1974), accounts for information processing instead of a storage-restricted task evoked by the idea of STM. Indeed, patients with STM impairment do not show LTM impairment, therefore STM is not always necessary for a LTM constitution. However, the idea of processing, and consequently of *levels-of-processing*, (Baddeley et al., 2002) again touches on the issue of meaning, without it taking a main role: “Hence, if the subject merely noted the visual characteristics of a word, for example whether it was in upper or lower case, little learning would follow. Slightly more would be remembered if the word were also processed acoustically by deciding, for example, whether it rhymed with a specified target word. By far the best recall, however, followed semantic processing, in which the subject made a judgment about the meaning of the word, or perhaps related it to a specified sentence, or to his/her own experience” (Baddeley et al., 2002, p. 5).

According to empiricism, the trace is in correspondence with the world. Similarly, according with computational theory of mind, LTM leads to autobiographical memory formation, and discordances between reality and memory are considered memory mistakes. The notion of “semantic” is avoided as a constitutive feature of the trace. Since this period, the term “semantic” has been used for “semantic memory” which “refers to memory for facts, including general knowledge about the world” (Manns and Squire, 2002, p. 82). It supports the cybernetic idea of information processing for a better adaptation, as it refers to the correspondence between the name of objects and their properties (Manns and Squire, 2002).

## The Age of Neurosciences: Multiple Traces Theory and Reconsolidation

Since the 1990s, neuroscience has been considered the best way of knowing human nature. In 1990, George Bush, the president of the United States of America, declared the start of the “Decade of the Brain.” Neurosciences, as if was an epistemological trend, took major proportions, becoming state policy in the United States of America. In the same vein, the Human Brain Project, aiming to allow an advance knowledge in neuroscience, computing, and brain-related medicine, started in 2013 including more than 20 countries. Thus, biology is the contemporaneous place to deposit anguish, as physics had once been (Ansermet, 2009), leading to important advances in the field.

Probably because of the new status of neurosciences, the correlation of anatomical localization and task–related–activation is used as evidence helping to describe trace. Indeed, the fact that traces stay temporarily in the hippocampus, and then migrate to the prefrontal cortex in order to become a LTM (Scoville and Milner, 1957; McClelland et al., 1995) is considered a support to the consolidation theory. Also, because the involvement of the hippocampus in the recovery of memories (Scoville and Milner, 1957) and in contextual memories, the semantic perspective returned into some researchers’ discourse (semantic memory understood as information obtained from the context allowing to better control the environment and adapt the response, this studies are mainly supported by experiments on mice). Then, in 1997, Nadel and Moscovitch proposed the “Multiple Traces Theory,” in which “the core notion is that episodic memory trace consists of linked ensembles of the hippocampal complex and neo-cortical neurons and that the hippocampal component remains a necessary part of that ensemble as long as the memory traces are viable” (Nadel and Moscovitch, 1997, p. 224). For these authors, the hippocampus rapidly organizes neuronal sets for all episodic information according to its semantic properties (Dudai et al., 2015).

According to this theory, the existence of the trace depends on semantic assemblies involved at the time of trace reactivation, that is, the semantic properties of the trace working as an index for neo-cortical neurons. Thus, new coherent representations are semantically structured oriented by the context. It is a new turning point in neurosciences. Rather than an associationism guided by simultaneity, it is a semantic associationism (in this context, “semantic” indicates context similarity), as Freud suggested with his Nachträglichkeit theory.

Over the last decade, a paradigm shift took place, extending the “Multiple Trace Theory.” The “fixation” idea was mostly abandoned giving rise to the reemergence of the idea of a malleability of the trace with the “reconsolidation” hypothesis (Dudai et al., 2015). The memory reconsolidation model, in continuity with the “Multiple Trace Theory,” suggests that the trace may be labile after its encoding. Experimental work, first on mice then on humans, has shown that several days after learning, if there is a confrontation to a reminding cue, the trace may be disrupted by an amnesic agent (Nader et al., 2000; Sara, 2000; Dudai, 2004; Alberini, 2011). The most frequently used amnesic agent is propranolol, a noradrenergic transmission blocker, whose role in memory consolidation is well documented (McGaugh, 2013). Additionally, no change in memory is reported when the amnesic agent is administered without the reactivation of memory.

Thus, memory reconsolidation is a form of plasticity with paradoxical aspects: a labile mechanism of memory after its reactivation (Nader, 2003), a process participating in memory stabilization (Alberini, 2005), and an increasing the strength and longevity of memory (Alberini, 2011). This model, still hypothetical, is at the core of an indissociable paradox of mnemonic functioning: a mechanism contributing to the subject’s identity and continuity, but also, a mechanism supporting a permanent change (Alberini et al., 2013).

In laboratory environments the reconsolidation mechanisms are described as a specific natural form easily marked by a temporal window varying to the type and age of the memory (Nader et al., 2000) and having molecular specificities that differentiate it to memory consolidation (Alberini, 2005). Although, throughout a human being’s life, there are continuous reminder cues of previous events, permanently opening the reconsolidation temporal window (Dudai and Eisenberg, 2004), whereby the mechanism of reconsolidation may be considered continuously activated in daily life (Dudai, 2012). According to this author, there would be only two utopian ways to not permanently reconsolidate: if the learning context is always exactly the same, or if the internal representations do not reactivate at all during learning (Dudai, 2012). Indeed, neither condition is the case in human experience.

**FIGURE 1:**
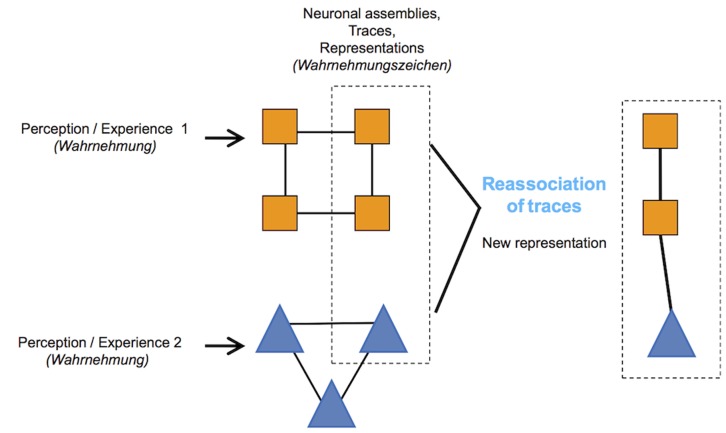
**The geometrical forms and colors indicate the different neuronal assemblies conforming the trace or parts of it**. The trace of Perception/Experience 1 and the trace of Perception/Experience 2 may lead to a new trace, a new representation, or a combination of elements from each.

On the basis of the human experience phenomenology, according to the Dudai’s insight, this theory hints at the permanent semantic reconstruction of the trace, suggesting discontinuity between the experience and its inscription through the permanent reassociation during production of new traces (Alberini et al., 2013; see **Figure 1**). Thus, there is a separation between the subject, or the lived experience, and the factuality of the experience.

This perspective highlights the plastic features of the human brain that allow for permanent reactivation, reassociation and inscription of experiences. If the trace is not over-determined by its inscription, it is then unpredictably determined by the plastic features of the brain. Moreover, this perspective posits that inscriptions may evolve, giving rise to the possibility of autopoiesis of the subject via the creation or production of new traces from the inaugural traces (Alberini et al., 2013).

Ben-Yakov et al. (2013) conducted an experiment broadening the scope of the semantic dimension in trace construction. Participants watched a narrative audiovisual clip (common daily routine scenes produced by an audiovisual production team working with the research team) while researchers used functional magnetic resonance imaging (fMRI). Hippocampal activation was explored by overlapping two clips (Clip_Clip vs. Clip_Fix, Clip_Scr), and the semantic dimension was explored by incongruence in the scrambled screen condition compared to the two presented clips (Clip_Clip vs. Clip_Scr). Immediately following the narrative clip (8 s in duration), one of three experimental conditions was presented: (1) to search coherence in a second narrative clip semantically unrelated to the first (Clip_Clip); (2) fixation, a red cross in the middle of a gray screen where participants fixate (Clip_Fix); (3) incongruence, a visually scrambled clip (pixels and background noise) (Clip_Scr). Next, memory was assessed 20 min after presentation of the narrative movie clip. The participants were previously informed about the memory test.

**FIGURE 2:**
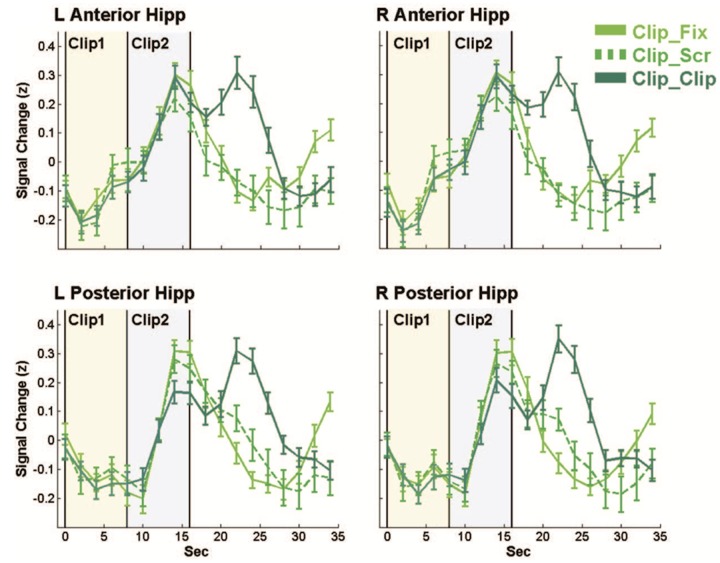
**Average of increasing activity in anterior (score *z*-scored data), posterior, left and right hemisphere through time**. The darkest green lines show a double peak hippocampus effect in the Clip_Clip condition as compared to single peak activation on the Clip_Fix and Clip_Scr conditions in light green. The first peak appears while the second clip has already started (gray zone) (reproduced with the kind permission of Y. Dudai).

The results showed that following presentation of the first clip, there was a hippocampal activation about 10 s after the end of the clip. The authors called this brain activation that is neither immediate nor direct response to the stimulus *offline activation*. Because the second clip was presented approximately at the same time as the hippocampal activation related to the first clip, the researchers proposed that there are two independent and alternating brain activations, *online* and *offline* (see **Figure 2**). Because the activation is not in direct response to the stimulus, the *offline* mode may be involved in an associative process. In this case, it may open a temporal gate of lability of old traces, as the memory reconsolidation model posits. New traces formed during the experience may then be structured under intrinsic activation following new associations with old traces.

There is then a continuous complex linkage between past and following experiences according to their semantic content in coherence with the “Multiple Traces Theory.” In this manner, this experiment hints at the diachronic dimension of the trace and supports an interconnection between the effort of building a semantically coherent autobiographical history and reconsolidation.

**FIGURE 3:**
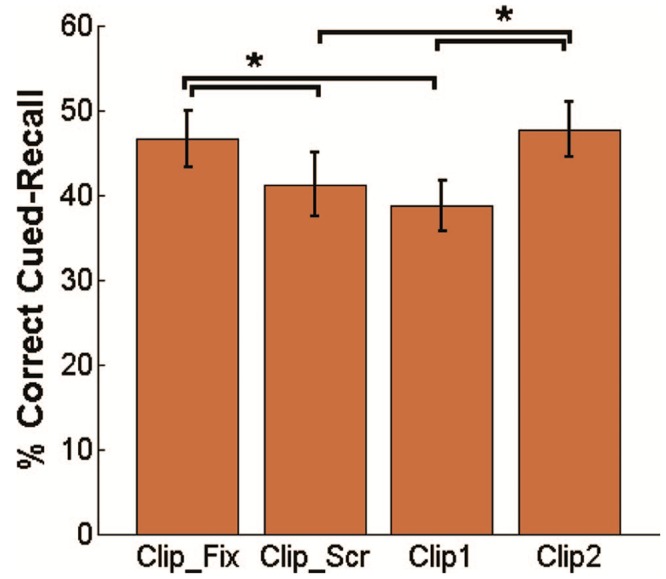
**On *Y*-axis: correct cued-recall in the test memory**. On *X*-axis: the clip assessed. The first clip (Clip1) of the Clip_Clip condition showed poorer recall in the memory test than the second clip (Clip2) in the same condition, and the clip in the Clip_Fix. The clip in the Clip_Scr condition shows poorer recall than for the clip in the Clip_Fix condition and for the second clip in the Clip_Clip condition (^∗^*p* < 0.05). Clip2 > Clip1 and Clip_Fix > Clip1 show a retroactive interference caused by the alternation between online and offline processes. Clip_Scr < Clip_Fix and Clip_Scr < Clip2 show a retroactive interference caused by the lack of meaning of the Clip_Scr condition (reproduced with the kind permission of Y. Dudai).

As the authors predicted, the results indicated better memory assessment of Clip 2 than for Clip 1 (see **Figure 3**). This is probably due to a more active role of the subject: participants may make an effort to find a link between the two clips and may find it easier in the case of the second, given that they already know what to expect. Similarly, there is a difference between the fixation condition and the scrambled screen condition (Clip_Scr < Clip_Fix). In our view, the failed attempt to understand the coherence given the lack of meaning of the scrambled screen leads to such differences. It was less disruptive for the subjects to fixate on a cross than to be confronted with the meaningless scrambled screen. Therefore, the fixation condition led to better conditions favoring trace construction than the scrambled screen. Again, the semantic dimension seems be an important factor for the trace construction.

This experiment brings a new insight in the study of the trace. It hints at another understanding differentiated from a computational account: the variable is not the time of processing meaningless information related to the memorization, but the differential meaning of the task. In this way, the mnesic trace may be understood by trace association and not by the time of processing or the control and processing of information. As such, this research explores in an ecological way the diachronic dimension, whereby the subject may reconsider past experience over time, and secondly, the attempts to build a meaningful experience, or semantic dimension, as a process that is not finalized at the end of the task.

## Phenomenology: Intrinsic Activity, Top-Down Models and Image

Another important philosophical trend is the phenomenology from which Merleau-Ponty highlighted in the 1960s the participation of the subject in the act of perceiving: the perceptual object in itself is considered a secondary construction, it is already a conceptual product of the encounter with the world and it is not an intrinsic feature of the object by itself (Merleau-Ponty, 1968). Thus, perception is not considered a univocal reality independent to the observer: “The thing, the pebble, the shell, we said, do not have the power to exist in face of and against everything; they are only mild forces that develop their implications on condition that favorable circumstances be assembled. But if that is so, the identity of the thing with itself, that sort of established position of its own, of rest in itself, that plenitude and that positivity that we have recognized in it already exceed the experience, are already a second interpretation of the experience” (Merleau-Ponty, 1968, p. 161).

Phenomenology points out that the human being constantly tries to anticipate perception in order to give a meaning to environmental information. Based on the work by Sokoloff et al. (1955), Raichle pointed out that the change of energy consumption in the brain associated with task performance is only 5% (Raichle, 2010). This underlines the importance of studying the other 95% of the activity, where the subject is not directly responding to the environment, but is rather generating intrinsic activity. This evidence has led to prolific investigation of the default mode of the brain (Buckner et al., 2008; Beaty et al., 2016) and hints at the relatively minor role of direct experience on overall brain activity (Qin et al., 2016).

Moreover, the quantity of information in our current environment is enormous compared to the filtered amount of information impoverished by the processing of our nervous system. For example, the retina can only process around 10^10^ bits/second. Due to the limited quantity of efferent axons, only approximately 6 × 10^6^ bits/second are emitted by the retina, from which only 10^4^ are reported to the afferent pathways (Raichle, 2010). Faced with a nearly unlimited amount of information in the world, the nervous system can only treat a small fraction of it; this information has then to be completed in order to establish coherence (Ansermet and Magistretti, 2010). Thus, more important than the quantity of environmental information is the choice of ancient traces that in a semantic consistency fill the blanks. Additionally, the study of the perceptive process concerns the anticipation process and the competition between hallucinatory pleasant traces and reality.

The opposition between environmental information processing and inner world construction is also reflected in the opposition between bottom-up and top-down theories. Bottom-up theories consider sensory information treatment as the combination of the features of the object allowing an objective, veridical, reconstruction of the object properties (Engel and Singer, 2001; Engel et al., 2001). Bottom-up theories are consistent with the empiricist’s view of the trace as an “impression which remains in the mind” and with Semon’s idea of the engram as a product of “nervous irritable tissue” (Semon, 1921, p. 12). Conversely, the top-down model proposes that previously inscribed traces shape perception; attentional and predictive processes influence on perception through thalamocortical networks in a determined temporal structure.

More specifically, the dynamic top-down theory proposes that populations of neurons synchronize in a network allowing other neuron populations to arise (Engel et al., 2001). This mechanism may form a larger inter-areal network (on visual recognition see Bar et al., 2006) by collateral association^11^ that may be in accordance with the social context or the meaning of the situation. Thus, there is a neuronal activation generated intrinsically, and not only as response to the stimulus. According to this view, the perceived object acquires a meaning that is not only intrinsic to the object but is also linked to the perceiving subject. Engel and Singer (2001) speculated that the mechanism underlying the synchronization, in gamma oscillations, of neuron populations might correspond to the reinterpretation of the item representation.

Paradoxically, although synchronic gamma oscillations have been experimentally related to the situation in which perception meets expectation, they have also been related to false memories (Buzsaki, 2006). This suggests that the predictor or expectation component may be as important as the perceptive phenomena, similar to the Freudian idea of a hallucinatory component in perception and the role of the semantic dimension in the construction of the trace.

This may be related to the Lacan’s perspective, a psychoanalyst in interaction with phenomenological thinking. For Lacan, the notion of memory as a set of neuronal spawning is insufficient if it is not introduced by the notion of image (Lacan, 1988 [1953–1954]). We understand “image” here as a representation of the “wishful activation” in Freudian terms related to hallucination, as presented above.

## Concluding Discussion: Syntax Vs. Semantic

This article organizes the history of trace theory in four schematized trends of thought through terms or notions associated to each trend of thought. For the sake of brevity these terms and notions do not correspond to an exhaustive list. Moreover, we schematized these four trends despite their blurred edges. Thus, *mneme*, engram, mark left by perception (*Wahrnehmungszeichen*) are related to the tension between empiricism and humanism. The notions of consolidation, learning and long term memory are related to cybernetics, around the 1940s–1950s. Multiple traces theory and the memory reconsolidation model are related to the rise of the neurosciences era, which extends until today. Finally, the notions of intrinsic activity, top-down models and image were related to the phenomenological tradition. In turn, these four trends of thought can be related, as mentioned in the “Introduction,” to two approaches of the study of memory highlighted by Goldstein (2011), that is, the *physiological approach* and the *mental approach*.

It is, however, more accurate to refer this two approaches to the study of memory to the division between structuralism and post-structuralism. Indeed, the statement “for the being who speaks and demands, the body fails the enjoyment inscription” (Laurent, 2016, p. 15) mentioned in the “Introduction,” according to the author, evokes post-structuralist thinking. Accordingly, it can be understood as a response to the structuralist considerations. This approach neglects the signification and the function issue, favoring the formal organization of the structure. Structuralists ideas aim to find universal rules of organization allowing to explain, and, to some extent, to predict human nature.

This is also a general intention of movements aiming to describe the trace physiology, which we relate here to Chomsky’s syntax conceptualization, since it generalizes the generative grammar as a formalization of rules allowing to predict the structure of production of new sentences (Chomsky, 2006). According to Chomsky, the human being is essentially a syntactic animal (Chomsky, 1957); he promoted the idea that the understanding of syntax, the inscription rules, allows us to better understand the functioning of the brain. This theory promoted the advent of the cognitive and neuroscientific era, and defended the idea that the human body can be understood as a biological machine predefined by its rules of functioning, what induce unfortunately, to an impoverishment in the function of language and meaning theorization.

Indeed, an important criticism to Chomsky was provided by the philosopher Searle (1990). He offered a relevant consideration concerning the semantic conditions of the trace with his idea of the “Chinese room.” In this thought experiment, a person who does not understand Chinese is in a closed room with a Chinese textbook. This person answers questions slipped to him under the door by people outside of the room who understand Chinese. He answers with a certain consistency and in a way that the people outside cannot know whether or not he speaks Chinese. The syntax is right, the signs and the ideas match, but he does not really understand Chinese. Hence, the understanding of a language and its meaning exceeds correspondence between the sign and the signified.

Searle’s argument is against the idea that consciousness is nothing more than a computer that produces signs and meanings of the signs (Searle, 1990). To Searle, consciousness is more than just the result of calculation sequences; it is rather an illusion given by semantic laws that create an independent reality to those who observe it and affected by the social context.

Indeed, the meaning gives the impression that a sign can occupy the assigned place, as if the signifier and the signified were linked: but the relation of meaning is arbitrary, the sound image (the signifier) seems to make an unambiguous reference to the object (the signified). This is why a differentiation is made between syntax and semantics. There is a detachment between the writing, reproduction of correspondences between the sign and the signified, and the reading (or trace reactivation), in which a creative process at work is multiplying the significations.

The syntax would then have an intrinsic impossibility to report these multiple readings of the trace (Jacob, 2003). However, does the writing – in every rewriting – ask the meaning to become undifferentiated? At best, the syntax seeks to report an effect of meaning that does not belong to it. It is what shows the constructed nature of the trace, and why we can say that writing is exactly what leads to the ambiguity (Laurent, 2016) and to reinvention. Despite the answers that can be provided from one or another trend of thought, the major question of this article is still whether the structure (in this context the syntax or the physiological mechanism description) may to account the meaning effects (with its necessary references to enjoyment). In so far that is the claim of post-structuralists, this article suggest that the post-structuralist argue is still a highly topical issue.

From Searle’s perspective, it seems that the inscription and the semantic dimension of the trace is far from being understood under a solipsistic explanation of the syntactic rules of inscription of the trace itself. His thought experiment makes the point that understanding meaning requires social convention, but also, that social inscription gives spoken language multiple interpretations, broadening the semantic inscription, and in turn, participating in the trace construction.

This does not imply that the syntactic model is solipsistic. The discover of mirror neurons in the 1980s and 1990s introduces the notions of empathy for example in a syntactic fashion. Indeed, intersubjectivity is conceptualized as an achieved information entering in the brain, being processed and used to better control the environment. Inputs are, in this line of thought, fully represented through the brain. Nevertheless, if according to Searle spoken language contributes to the semantic dimension (and it cannot be dissociated from the syntactic level touched on by diachrony), there remains an open question about the role of social context in forming a semantic inscription on the trace over time.

From an ontological perspective, our conclusion from this historical review is that the reconstructive nature of the trace, which is permanent, is at the service of the subject’s poiesis. From an epistemological and historical perspective, our conclusion is that notwithstanding the important influence of empiricism in the study of the trace, the humanistic or more ecological or phenomenological commitments frequently nuanced the empiricist position.

Therefore, if diachrony and semantics are intertwined, this implies that meaning processing is never really completed, despite the trace syntax or correct engraphy, as if rewriting follows the meaning. The potentialities of creation seem constantly tested by the lack of meaning to which the subject is confronted in his life. The subject has then an active and permanent role in the construction of the trace, giving rise to a diachronic dimension of the trace. From this perspective, instead of being a pure brain reaction to the environment, new trace constitution depends on the subject’s search for meaning.

From a psychoanalytic perspective, this has clinical implications that have already been pointed out by Lacanian psychoanalysis, whereby the psychoanalyst “has to proceed as if the signifier of the construction had the same value as the analysand’s memory” (Miller, 2010, p. 8), because “what is historical truth? It’s not the exactitude of what took place, it’s the reorganization of what took place through the perspective of what will be” (Miller, 2010, p. 11).

Although this discussion does not allow for a comprehensive review of the finer details and analysis of the phenomenology of human inscription of experience, we highlight that the rich differences in experiences, and consequently, the rich differences in their inscription must involve an infinite variety in the modes of brain activity during trace inscription, particularly because the search of meaning of each experience may be as infinite as diachrony of the trace may be. Hence, we defend the idea that the inscription of the trace cannot be investigated as a unitary construct, given that the last version of meaning is not accessible.

## Author Contributions

CE is the main contributor of this paper as part of her Ph.D. thesis. PM and FA, as supervisors, contributed to the conception and development of the research, and they revised critically the manuscript for intellectual content.

## Conflict of Interest Statement

The authors declare that the research was conducted in the absence of any commercial or financial relationships that could be construed as a potential conflict of interest.

## Footnotes

1 “Il est même impossible de ne pas voir ce qui frappe nos sens” it is impossible not to see what hits our senses (our translation, Nicole, 1709 [1675], p. 87).

2 “Est synchronique tout ce qui se rapporte à l’aspect statique de notre science, diachronique tout ce qui a trait aux evolutions,” it is synchronic all that relates to the static aspect of our science [linguistics], diachronic all that relates to evolutions of the language (our translation, de Saussure, 1997 [1916], p. 117).

3 “Il est même impossible de ne pas voir ce qui frappe nos sens” it is impossible not to see what hits our senses (our translation, Nicole, 1709 [1675], p. 87).

4 Associationism groups together several philosophical proposals in which the general idea is that the association of the mental states (perceptions, ideas) originates mental process as thinking. More precisely for Locke, the tendency to associate ideas informs about thinking in the human beings. It should not be confused with “free association” (in Freud’s German *freie Assoziation*), a method used in the psychoanalytical technique in which the patient is prompted to say everything that comes into his mind.

5 “[Humanism] offers an ethical point of view in which humans, residing in a secular world, might determine their own meanings and judge their own actions in service to a self-determined life (Tauber, 2009, p. 39).

6 “We know that of the percepts [*Wahrnehmungen*] which act upon the system W [consciousness-perception], we retain permanently something else as well as the content itself. Our percepts prove also to be connected with one another in the memory, and this is especially so if they originally occurred simultaneously [*Gleichzeitigkeit*]. We call this the fact of *association* [*Assoziation*]” (Freud, 2010 [1900], p. 537).

7 Faced with an external stimulus, the living organism tries to keep the arousal level as low as possible. The pleasure principle is then an avoidance of displeasure.

8 “Thus the experience of satisfaction leads to a facilitation between the two memory-images [of the object wished-for and of the reflex movement] and the nuclear neurons which had been cathected during the state of urgency. (No doubt, during [the actual course of] the discharge brought about by the satisfaction, the quantity *(Q*ǹ*)* flows out of the memory-images as well.) Now, when the state of urgency or wishing re-appears, the cathexis will pass also to the two memories and will activate them. And in all probability the memory-image of the object will be the first to experience this wishful activation. […] I have no doubt that the wishful activation will in the first instance produce something similar to a perception – namely, a hallucination. And if this leads to the performance of the reflex action, disappointment will inevitably follow.” (Freud, 1953 [1895], p. 381).

9 The term “hallucinatory” in the Freudian theory may be understood as a large phenomenon with gradations. Here, it is not a psychopathological sign, rather it is close to a “flashback” or revivification episode.

10 Contrary to common belief, the phrase summarizing the Hebbian postulate “cells that fire together wire together” is not Hebb’s, but rather Shatz’s (1992).

11 The term “association” may not be confused with the notion of “free association.” Rather, it is conceptually close to Freud’s definition of “memory” (1962 [1896]) as “chains of associations”.
